# Supplementary material for: A Humanized Yeast Phenomic Model of Deoxycytidine Kinase to Predict Genetic Buffering of Nucleoside Analog Cytotoxicity
Source: Genes (Basel). 2019 Sep 30;10(10):770. doi: 10.3390/genes10100770 (PMC6826991; doi:10.3390/genes10100770)
Supplement: Supplementary file 1 [file genes-10-00770-s001.zip › Additional_Files/Additional_File7_GOTermHeatmaps.zip!/Additional_File7_GOTermHeatmaps/maturation_of_SSU-rRNA.pdf]

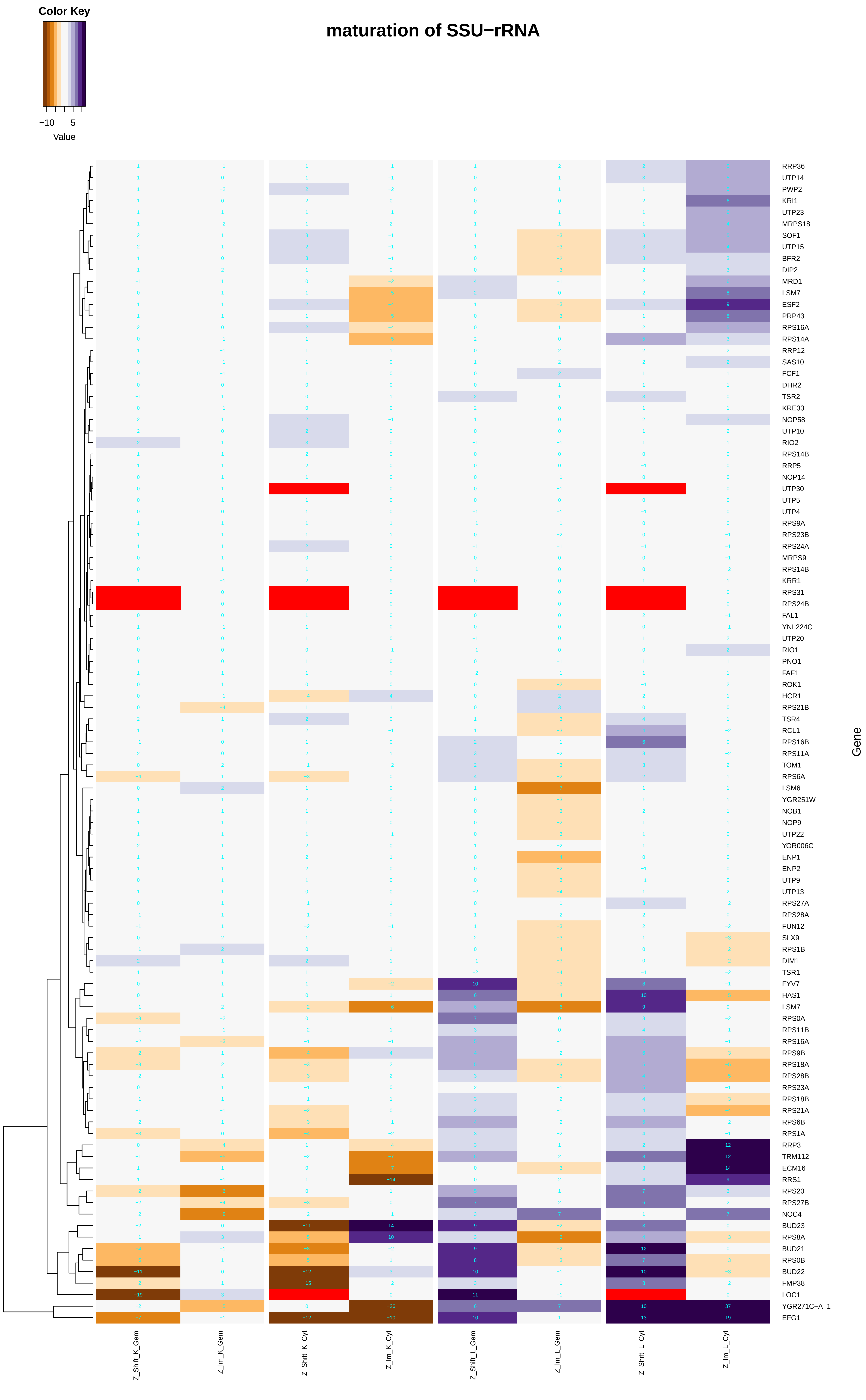
maturation of SSU−rRNA
Color Key
−10
5
Value
Gene
RRP36
UTP14
PWP2
KRI1
UTP23
MRPS18
SOF1
UTP15
BFR2
DIP2
MRD1
LSM7
ESF2
PRP43
RPS16A
RPS14A
RRP12
SAS10
FCF1
DHR2
TSR2
KRE33
NOP58
UTP10
RIO2
RPS14B
RRP5
NOP14
UTP30
UTP5
UTP4
RPS9A
RPS23B
RPS24A
MRPS9
RPS14B
KRR1
RPS31
RPS24B
FAL1
YNL224C
UTP20
RIO1
PNO1
FAF1
ROK1
HCR1
RPS21B
TSR4
RCL1
RPS16B
RPS11A
TOM1
RPS6A
LSM6
YGR251W
NOB1
NOP9
UTP22
YOR006C
ENP1
ENP2
UTP9
UTP13
RPS27A
RPS28A
FUN12
SLX9
RPS1B
DIM1
TSR1
FYV7
HAS1
LSM7
RPS0A
RPS11B
RPS16A
RPS9B
RPS18A
RPS28B
RPS23A
RPS18B
RPS21A
RPS6B
RPS1A
RRP3
TRM112
ECM16
RRS1
RPS20
RPS27B
NOC4
BUD23
RPS8A
BUD21
RPS0B
BUD22
FMP38
LOC1
YGR271C−A_1
EFG1
Z_Shift_K_Gem
Z_lm_K_Gem
Z_Shift_K_Cyt
Z_lm_K_Cyt
Z_Shift_L_Gem
Z_lm_L_Gem
Z_Shift_L_Cyt
Z_lm_L_Cyt

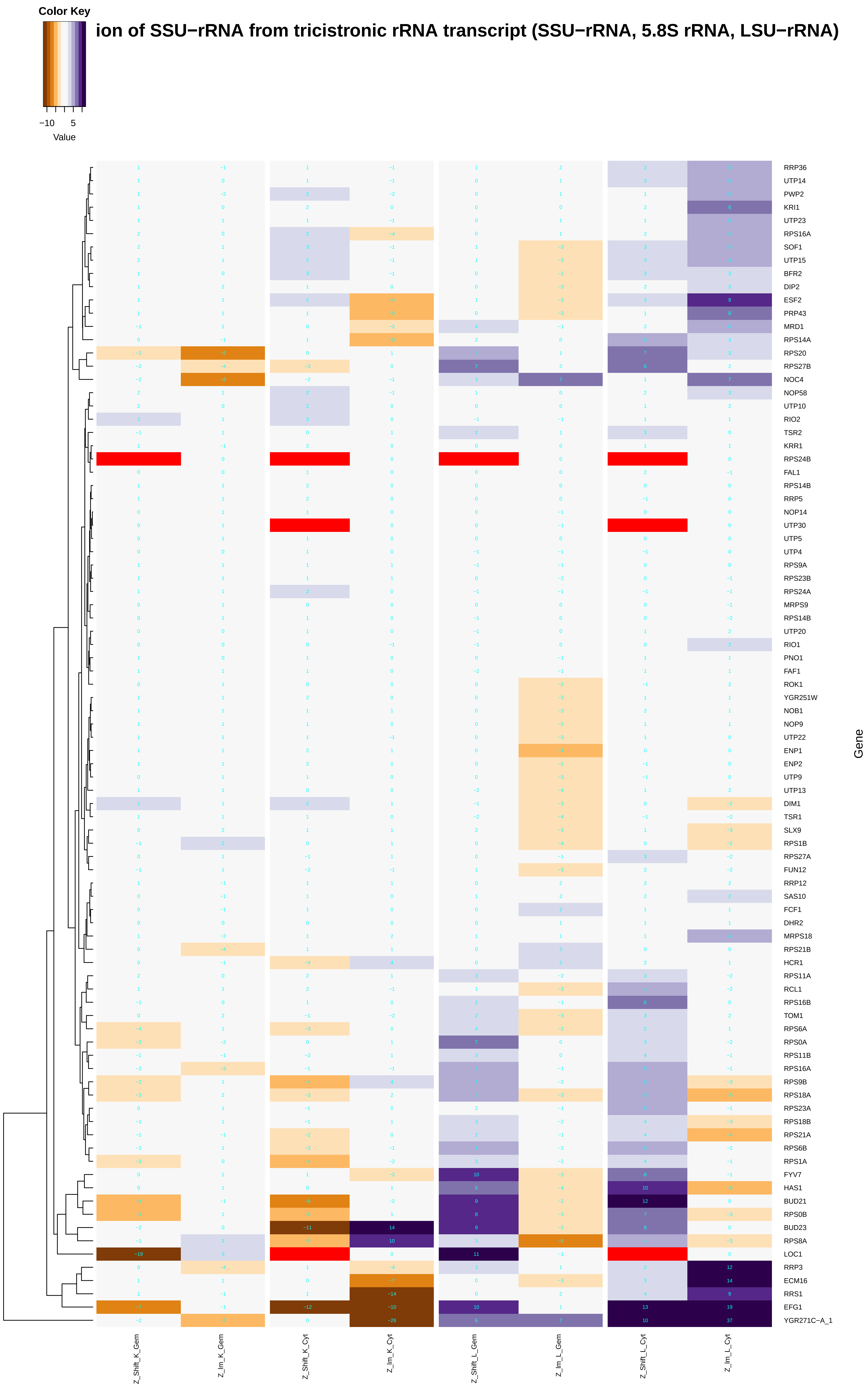
Color Key
−10
5
Value
ion of SSU−rRNA from tricistronic rRNA transcript (SSU−rRNA, 5.8S rRNA, LSU−rRNA)
RRP36
UTP14
PWP2
KRI1
UTP23
RPS16A
SOF1
UTP15
BFR2
DIP2
ESF2
PRP43
MRD1
RPS14A
RPS20
RPS27B
NOC4
NOP58
UTP10
RIO2
TSR2
KRR1
RPS24B
FAL1
RPS14B
RRP5
NOP14
UTP30
UTP5
UTP4
RPS9A
RPS23B
RPS24A
MRPS9
RPS14B
UTP20
RIO1
PNO1
FAF1
ROK1
YGR251W
NOB1
NOP9
UTP22
ENP1
ENP2
UTP9
UTP13
DIM1
TSR1
SLX9
RPS1B
RPS27A
FUN12
RRP12
SAS10
FCF1
DHR2
MRPS18
RPS21B
HCR1
RPS11A
RCL1
RPS16B
TOM1
RPS6A
RPS0A
RPS11B
RPS16A
RPS9B
RPS18A
RPS23A
RPS18B
RPS21A
RPS6B
RPS1A
FYV7
HAS1
BUD21
RPS0B
BUD23
RPS8A
LOC1
RRP3
ECM16
RRS1
EFG1
YGR271C−A_1
Gene
Z_Shift_K_Gem
Z_Im_K_Gem
Z_Shift_K_Cyt
Z_Im_K_Cyt
Z_Shift_L_Gem
Z_Im_L_Gem
Z_Shift_L_Cyt
Z_Im_L_Cyt
